# If You Build It Will They Come? Park Upgrades, Park Use and Park-Based Physical Activity in Urban Cape Town, South Africa—The SUN Study

**DOI:** 10.3390/ijerph20032574

**Published:** 2023-01-31

**Authors:** Clare A. Bartels, Estelle V. Lambert, Marié E. M. Young, Tracy Kolbe-Alexander

**Affiliations:** 1 Research Centre for Health through Physical Activity, Lifestyle and Sport (HPALS), Division of Physiological Sciences, Department of Human Biology, Faculty of Health Sciences, University of Cape Town, Cape Town 7700, South Africa; 2 Department of Sport, Recreation and Exercise Science, Faculty of Community and Health Science, University of the Western Cape, Cape Town 7535, South Africa; 3 School of Health and Medical Sciences, University of Southern Queensland, Ipswich, QLD 4305, Australia; 4 Centre for Health Research, University of Southern Queensland, Ipswich, QLD 4305, Australia

**Keywords:** physical activity, parks, SOPARC, Cape Town, South Africa, low-middle income country

## Abstract

The development and upgrade of recreational public spaces are key government strategies to increase opportunities for physical activity (PA) and enhance social interaction and community cohesion. This study aimed to evaluate differences in park use and park-based PA in recently upgraded/developed parks (intervention, *n* = 4) against established parks (control, *n* = 4) and in regional parks in high- and low-income settings (*n* = 2). Additionally, associations between target area features, park use and PA were identified. Direct observation of park use and attributes was conducted using the System for Observing Play and Recreation in Communities (SOPARC) over four months. Despite more park users in intervention parks (2519 vs. 1432), control park visitors were 48% more likely to be engaged in PA (*p* < 0.001). Similarly, while high-income park users attracted more visitors (2135 vs. 1111), they were 79% less likely to be engaged in any PA compared with low-income park visitors. The likelihood of both use of and PA by gender and age differed by features. Active recreation features in intervention parks attracted more users than the same features in control parks. In this study, upgraded or newly developed parks attracted more visitors but not necessarily overall greater levels of physical activity.

## 1. Introduction

The spatial distribution of built environment opportunities for physical activity impacts on health outcomes and quality of life [1,2]. Previous research has shown that a higher number of and improved quality of parks and recreational facilities are associated with increased levels of physical activity [3] and a lower prevalence of both type 2 diabetes [4] and obesity [5]. Empirical studies have also associated parks with greater health and wellbeing [6] and improved social interactions [7,8,9]. Furthermore, physical activity in the form of play is considered an essential part of childhood development [10]. Thus, green public spaces such as parks are an integral resource for communities as they provide access to a wide range of activities and potential health benefits [11,12,13]. For lower-income communities these benefits may be particularly pertinent due to the burden of disease [14].

However, the inequitable distribution of park and recreation facilities among populations from different socio-economic settings may also impact on physical activity behaviour. Those living in lower socio-economic communities tend to have less access to parks and recreational facilities, and where they do exist, are often of lower quality. Often, high crime rates and greater threats to personal safety are additionally experienced [15]. As a consequence, they may engage in less recreational physical activity [16,17,18,19,20,21,22]. This is particularly relevant to South Africa where 57% of the population is failing to meet the global physical activity recommendations [23].

Factors such as park design and park features, as well as access and proximity, have been shown to influence park use and park-based physical activity [23,24,25,26,27]. In order to address issues of environmental injustice and to provide opportunities for community-based physical activity, the renewal, development and maintenance of parks and recreational facilities are receiving substantial investment by governments globally [28], particularly in high-density, low-income residential neighbourhoods. This has been shown in Victoria, Australia, where the renovation of a large regional park in a low-income neighbourhood resulted in a significant increase in park use and park-based physical activity [29].

The spatial disparity created in South Africa during apartheid (1948–1994) resulted in widespread inequality, as access to quality and well-serviced public amenities was based on race [30]. This legacy remains, as residents in the highest density and lowest income areas in Cape Town continue to have the least access to park and recreational spaces [31]. The City of Cape Town municipality (hereon called ‘City’) has therefore invested considerable public funds into recreational facility development and park upgrades [City of Cape Town, personal communication]. One of the City’s investment projects is the Smart Parks project, which involves a principle-based approach to the planning and delivery of parks. The set of principles relate to the choice of park location, integration with community facilities, the delivery of high-quality sustainable facilities and community consultation [32].

Taking into consideration the factors that influence park use as well as the City’s commitment, the evaluation of new park developments and upgrades is important in order to inform policy and planning and establish priorities to promote physical activity. These evaluations can provide information on park user demographics, park users’ activities at the park, and the extent to which improvements to park facilities result in increased park use and physical activity levels [33,34,35].

Despite the growth in evidence over the past decade, there are no empirical studies reporting the association between parks and park facilities on physical activity behaviour from the African continent. An even greater dearth exists evaluating these large-scale investments on physical activity behaviour. The primary aim of this study is to document park use and park-based physical activity in relation to recreational park features and settings in 10 urban parks in Cape Town, South Africa. The study objectives include:

(1) Evaluate the differences in park use and park-based physical activity between one large upgraded park with one income-matched control park as well as three newly developed community parks with three geographically matched parks (Objective 1);
(2) Evaluate the differences in park use and park-based physical activity between two large integrated regional recreational parks in a low-income and high-income setting (Objective 2);
(3) Determine the association between target area features, park use and park-based physical activity across these 10 parks (Objective 3).

## 2. Materials and Methods

### 2.1. Research Design and Setting

This study evaluated natural interventions using a cross-sectional, post-test design involving direct observations of park characteristics and park visitors in (1) eight parks in five lower-income suburbs, representing four intervention and four control parks (Objective (1) and (2) two newly developed integrated regional recreational parks, one in a low-middle income and another in a high-income suburb (Objective 2). The City Parks Department designed, developed and managed these parks. In 2017, City Parks merged with the Sports, Recreation and Amenities Department to become the Recreation and Parks Department.

Written authorisation to conduct the study was provided by the City and ethical approval was granted by the University of Cape Town’s Human Research Ethics Committee (HREC number 310/2015).

The City’s Parks Development Policy describes different categories of parks. Our study identified all park categories for inclusion, including the only two regional parks, two district parks, three community parks and three community Smart Parks (Table 1).

**Figure 1:**
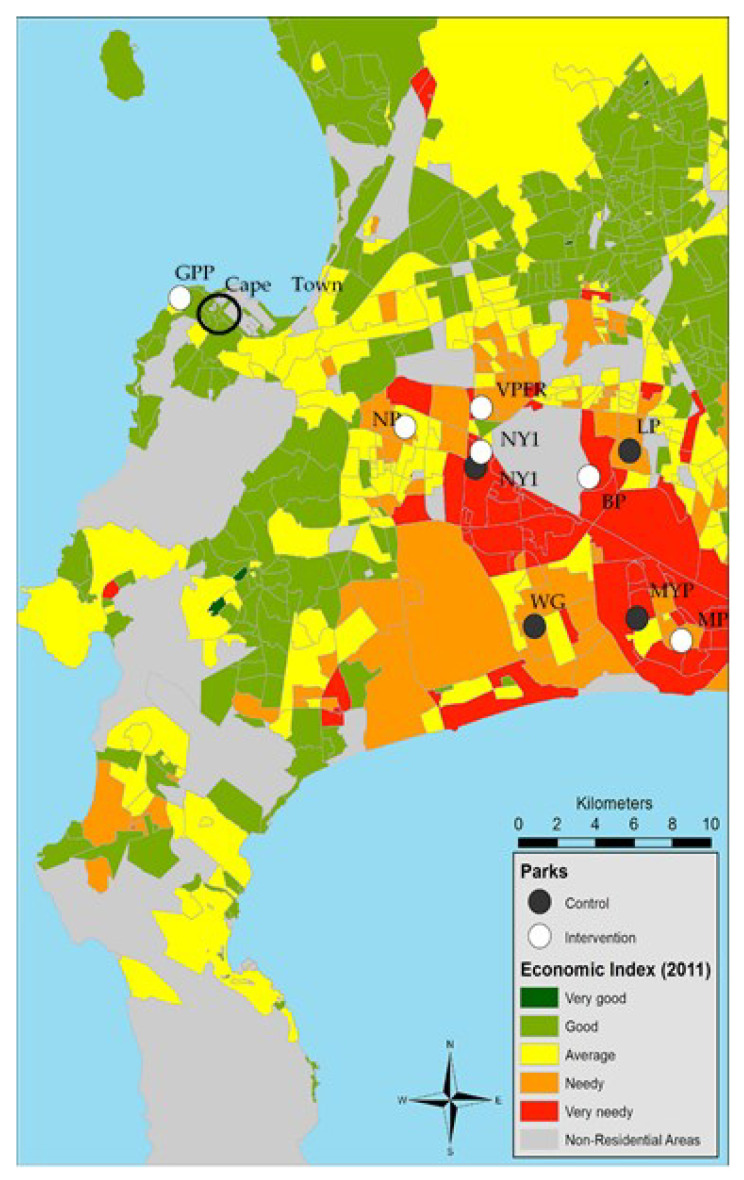
Park location by Economic Index for the city of Cape Town. Note. White-intervention parks (GPP = Green Point Park—higher income suburb, VPFRC = Valhalla Park Family Recreation Centre—lower income suburb, NP = Nantes Park, BP = Blikkiesdorp Park, MP = Mandela Park), black-non-upgraded parks (LP = Longmead Park, WG = Westridge Gardens, MYP = Manyanani Park, NY 144 Park). Source. Information and Knowledge Management Department, City of Cape Town, 2014.

Selection of the upgraded or developed parks was based on their location within lower-income suburbs. At the time of the study, the intervention parks represented the only parks in the city that had recently been upgraded or newly developed. Comparison parks were existing parks that had not undergone an upgrade and were either located in the same geographical area (same suburb) or were income-matched (not located in the same suburb). Income status was derived from the most recent available data (2011 Cape Town Census) in which each sub-place (sub-division of a suburb) within Cape Town was allocated a socio-economic score, comprising of the household services index, the education index, the housing index and the economic index. For the purposes of this study, the economic index was used (the sum of employment, income and economic dependency ratio) (City of Cape Town, 2014, personal communication). A high-economic sub-place (area) is hereafter referred to as high-income and low-economic sub-places are referred to as low-income (Figure 1). The study sample comprised of all park users in the *intervention and control* parks.

#### 2.1.1. Intervention and Comparison Parks (Objective 1)

##### Upgraded Park vs. Non-Upgraded District Parks

Nantes Park is an upgraded district park located in a low-middle-income suburb. The park is one of 13 district parks in Cape Town and was the only district park receiving upgrades at the time of the study. The park is over 40 years old, and due to neglect and vandalism, it became a hub for illegal activities and frequent dumping. A rejuvenation process of Nantes began in 2007 after lobbying and requests by the surrounding communities, and with the support of the City of Cape Town. The process and steps were undertaken with the community aligned with the City of Cape Town’s Public Engagement Policy of 2009. The full restoration was completed in 2013. The 17-hectare-sized park, after upgrade, included separate play areas for different age groups, shaded and seated picnic area, a skate park, outdoor gym, amphitheatre and fencing around the perimeter to increase safety.

The income-matched comparison district park was Westridge Gardens. The 14 hectare-sized park consists mainly of green open areas but also contains a gravel play area and a skate park. Funding was received to upgrade the park in 2010; however, this was for the establishment of a rose garden, completed in 2011. An outdoor sports precinct is situated adjacent to the park. The suburb had the highest reported incidence of crime in South Africa (http://www.crimestatssa.com, accessed on 19 October 2016) at the time this study was conducted.

Both parks are located in a residential area, have similar demographic characteristics and socio-economic status (City of Cape Town 2011 census). Furthermore, both parks are frequently utilised for community concerts, festive programmes, or hired for events and smaller functions by the local community. Nantes Park and Westridge Gardens serves as a park-pair comparison between an upgraded park (intervention) and income-matched non-upgraded park (comparison).

##### Community Smart Parks vs. Control Parks

The Symphony Way Smart Park (known as Blikkiesdorp Park, as the park is situated in the informal settlement of Blikkiesdorp), NY110 Smart Park and Mandela Smart Park represented the only three community Smart Parks in Cape Town at the time of the study and were newly developed between 2014 and 2015. For the City to respond to future growth needs, it undertook scientific modelling, consultation with internal City stakeholders and an evidence-based approach to identify the most underserved and priority investment areas for social facilities and public spaces. The City’s Capital Investment Framework also guided park development and upgrades in the most underserved communities. The aim was to enable equitable access, to develop high quality, unique and user-friendly parks that responded to the recreational needs of the served communities, and to contribute to integrated public investment and promote partnerships. Once the suburbs were identified, key City Parks Department operational staff were consulted to further refine the locations for potential Smart parks. Thereafter, the City’s Community Services Portfolio Committee, which includes elected councillors representing communities, was consulted to finalise agreement and approval for the first three neighbourhoods. The final step was engaging with members of each of the selected communities to identify the most suitable site from the list of proposed sites. Once the final site was selected together with the community, the co-planning and design of the park ensued (personal communication, Head Planning and Development, Community Services and Health, City of Cape Town).

The three Smart parks are located in low-income suburbs and each contains a play area, multi-purpose court for sports such as soccer, and a seated picnic area. Additionally, the NY110 and Mandela parks contain an outdoor gym, green open areas, a netball/basketball court and a small-sized, synthetic, multi-purpose pitch. Each of the three parks were matched with a control community park of similar size located within 2.5 km Euclidian (straight line) distance, namely Longmead Park, NY144 Park and Manyanani Park, respectively. These parks have not undergone upgrade, other than general repair and maintenance.

#### 2.1.2. Integrated Recreational/Regional Parks (Objective 2)

The Green Point Urban Park (GPP) and Valhalla Park Family Recreation Centre (VPFRC) represent two large integrated regional recreational parks and serve as a park-pair comparison between a high-income and lower-income suburb. The two parks also represent the City’s only regional-level parks. Green Point Urban Park is a 12.5-hectare sized park situated in a high-income suburb, located approximately 3 km away from the Cape Town CBD. After South Africa was awarded the 2010 FIFA World Cup, the precinct was re-designed and opened in 2011 [36] to include formal and informal sport and leisure facilities. These include play parks, a golf-course, tennis courts, rugby fields, football fields, the Cape Town Stadium, the Green Point Athletics Stadium and a private indoor gym. These facilities are adjacent to the park. The park attracts visitors from across the metro and beyond due to its aesthetic nature and partly due to its surrounding public transport and non-motorised transport route links.

The Valhalla Park Family Recreation Centre is located in a low-middle-income suburb on the Cape Flats region, approximately 14 km from the Cape Town CBD. The park, completed in 2013, is six hectares in size. At the time this study was conducted, the area and surrounding suburbs were reported to have the 7th highest crime rate in the Western Province (http://www.crimestatssa.com, accessed on 19 October 2016). Both GPP and VPFRC have separate play areas for different age groups, an outdoor gym and open green space. Additionally, VPFRC has a full-sized synthetic soccer field and informal active recreation areas including a skate park, multi-purpose courts and water spray zone.

#### 2.1.3. Park Features, Park Use and Park-Based Physical Activity (Objective 3)

Empirical data collected from the 10 parks (Objectives 1 and 2) were aggregated to determine the associations between the use of the different target area features (i.e., play areas, multi-purpose courts, etc.) by gender and age, as well as the associations between physical activity and target area features by gender and age. Table 2 provides a comparison of the recreational features of each intervention and control park.

### 2.2. Direct Observation of Park Characteristics and Park Users

The System for Observing Play and Recreation in Communities (SOPARC) was used to collect quantitative data on park use and park-based physical activity. SOPARC was selected as its outcomes aligned with the primary aim of the study—to obtain information on community park use and park-based physical activity by different users. The SOPARC tool is a validated and reliable tool [37,38,39] that employs systematic observations consisting of clockwise scans in pre-determined target areas. Target areas are specific areas in the parks that create opportunities for physical activity, such as play areas and green open spaces. Each individual was observed in the park, and the following measurement indicators were recorded:

- Period of the day: Morning, late morning, early afternoon, late afternoon
- Day of the week: Weekday or weekend
- Age group: Child (≤12 years), teenager (13–20 years), adult (21–59 years), senior adult (≥60 years)
- Gender: Male, female
- Physical activity level: Sedentary, walking, vigorous
- Location in the park (target area)

‘Sedentary’ was defined as any individual observed lying down, seated or standing in place. ‘Walking’ was defined as individuals walking at a casual pace. ‘Vigorous’ was defined as an activity greater than an ordinary walk [40]. In the current study, each individual’s gender, age group, activity level and activity were recorded simultaneously.

Seven park characteristics were recorded at the start of each scheduled scan for each target area:

- Accessible: Not locked or rented to others
- Supervised: By park staff
- Organised activities: Offered by park staff
- Equipped: Loose, non-permanent equipment
- Usable: Not excessively wet or windy, physical activity can be performed
- Dark
- Empty

The tool was revised to obtain additional park characteristics including family and social groups, the presence of dogs, general care and maintenance of the park and fencing. As the tool had not previously been used in the South African context, six of the 12 sport-related activity codes were replaced with the most populous sporting activities in South African urban settings, including netball, tennis, cricket, hockey, rugby, martial arts/karate and softball [41].

Eight observers were trained to conduct SOPARC. Training comprised of one day of theory as well as practical training utilising the online SOPARC videos. Thereafter, one practical session was conducted in the field to familiarise observers with the SOPARC tool. Each park was then visited to determine the target areas, coding stations (location from which scans were conducted), observation sequence (sequence in which the scans were conducted) and to further practice observations in the respective parks. Park managers were contacted prior to the initial visit to inform them of the study. Observers in the three low-income area Smart Parks were accompanied by two Neighbourhood Watch members who provided safety for observers while in the park.

Observations were conducted between the months of July and October 2015, representing winter (July to August) and spring seasons (September to October). On the day of scheduled observations, pairs of observers were equipped with the data recording sheets, pre-determined printed maps, clipboards and pens. Teams arrived at the park a few minutes earlier to rehearse the observation sequence. Intervention and comparison parks were evaluated simultaneously over a period of four days (consisting of four one-hour observation periods per day: 08:00, 10:00, 13:00 and 16:00). Observation times were based on available daylight and safety reasons. Observations were conducted on two randomly identified weekdays and two weekend days. According to Cohen et al. [42], 12–16 h of observations over a period of one week produces sufficiently robust estimates, similar to that found from 96 hourly measurements taken over a week. Observations were not conducted during inclement weather conditions. The average minimum and maximum temperatures recorded were 9° Celsius and 22° Celsius, respectively [South African Weather Service, personal communication, 23 November 2017].

### 2.3. Target Area Characteristics

A total of 136 target areas were pre-determined across the 10 parks. The number of target areas in each park varied according to the size of the park, ranging from 5–22 target areas. Observations were conducted across the entire park in seven parks. In the remaining three parks, the areas that were not observed included a biodiversity garden and large pond (*n* = 1 park) and unmaintained grass areas (e.g., long grass) (*n* = 3 parks). Target areas were classified using four main categories: (1) green open areas—these included grass areas for active and passive recreation, (2) play areas—playground hub with a variety of play equipment, (3) informal active recreation areas—including outdoor gyms, skate parks, multi-purpose courts and BMX a track, and (4) formal sports areas—synthetic soccer field, cricket pitch and netball/basketball courts. Grouped target areas were called target area features. The area size (m^2^) of each park was determined by the City of Cape Town’s online interactive Map Viewer.

### 2.4. Statistical Analysis

Data were evaluated using Stata statistical software (version 13.0). Users per target area, per time period and per park were calculated to obtain total number of users, number of users by gender and age group, users engaged in sedentary, walking and vigorous activity and the number of users per target area. Descriptive statistics describe park characteristics, park visitor demographics, park-based physical activity and physical activity area usage across all 10 parks as well as in sub-groups parks—intervention and control parks, and high-income and lower-income. The two main outcomes of interest were (1) the total number of park users observed across the four days and per observation period [43] and (2) the number and proportion of users engaged in physical activity (PA). Park users per observation period were calculated by the total number of people observed/number of observation periods. PA consisted of walking and vigorous physical activity.

Chi squared tests were conducted for categorical variables to determine the association between park visitor characteristics and park use between the intervention and control parks (Objective 1) as well as the high-income and low-income parks (Objective 2).

Logistic regression tests were conducted for each of the three objectives. For the first objective, we determined the association between physical activity behaviour (any PA vs. sedentary) and park user characteristics for intervention and control parks. This included comparisons between sub-groups (upgraded district vs. control, Smart community parks vs. control, and Smart community parks vs. upgraded district). For the second objective, we use the same analysis to compare high- and low-income parks. For the third objective, logistic regression was used to determine the association between park use and target area features (independent variables) by gender and age (dependent variables), as well as physical activity and target area features (independent variables) by gender and age (dependent variables). Univariable logistic regression models controlled for age (child), gender (male), period of the day (morning), period of the week (weekend), average temperature and wind speed. Significant variables were modelled in the final analysis.

## 3. Results

### 3.1. Overall Findings in the 10 Parks

Over a period of 40 days, a total of 7197 park visitors were observed in a cumulative area of 324,995 m^2^ across the 10 parks. In the high-income and low-income regional parks, only three scans were completed on one weekend day. Furthermore, protest action at Symphony Way Park (Blikkiesdorp Park) adversely affected observations on one week-day which prevented a fourth scan. This amounted to 157 observation points across the 10 parks. Green Point Park (high-income regional park) visitors accounted for 29.7% of all observations (*n* = 2135), Valhalla Park Family Recreation Centre (low-income regional park) accounted for 15.4% (*n* = 1111) while the two district parks Nantes Park and Westridge Gardens accounted for 14.2% (*n* = 1024) and 11.3% (*n* = 820), respectively, of all observations. The three community Smart parks accounted for 20.8% of all observations (Mandela Smart Park, *n* = 604; NY110 Smart Park, *n* = 525; Blikkiesdorp Smart Park, *n* = 370). The three non-upgraded community parks (Longmead Park, *n* = 145; NY144 Park, *n* = 226; Manyanani Park, *n* = 247) when aggregated, accounted for the fewest observations (8.6%).

Target areas in all 10 ten parks were accessible and usable, except for two parks, where a section of one target area was waterlogged. VPFRC was the only park that provided loose equipment such as balls, and supervised and organised activities. Family and social groups were observed most frequently in the upgraded parks as well as Westridge Gardens (the non-upgraded district park). All 10 parks were well maintained (regular grass cutting, watering or litter removed), but none of the parks had garbage bins. This is by design that visitors take their litter with them when they leave. Despite fencing and security in VPFRC, the toilets and indoor community centre had been vandalised and were closed off. Dogs were present in 19 observations, most of which were observed in Nantes Park, the upgraded district park.

Park visitors were mostly male (56.4%), and children (53.2%), followed by adult visitors (30.3%). Teenagers represented 16.4% of observations and only 13 senior adults were observed in all 10 parks, during the late morning and afternoon periods. No visitors with disabilities were observed. Parks were visited more frequently on weekends (68.8%) than weekdays and in the early afternoons (41.3%) than at the other times of the day. The majority of park visitors observed (62.7%, *n* = 4506) were engaged in sedentary activities such as sitting or lying down (e.g., relaxing, picnicking or sitting on play equipment) or standing (e.g., overseeing children in the playground). Twenty-one percent were observed walking and 14.6% were engaged in vigorous-intensity activities. Structured pathways were not used for exercise but rather for traversing the park to reach target areas or as a general thoroughfare.

### 3.2. Intervention and Comparison Parks (Objective 1)

In total, 3951 park visitors were observed in the eight parks over a period of 32 days, of which 2519 (63.8%) were observed in the intervention parks (*n* = 4). On average, 111 visitors were observed per hour in the week and 204/h. on the weekend in the intervention parks. In the comparison parks (*n* = 4), an average of 54 visitors were observed per hour in the week, and 25/h. on the weekend. There was no difference in the proportion of males and females between intervention and comparison parks, however children were more likely to be observed (*p* < 0.001) than adults in intervention parks.

While both intervention and comparison parks had more weekend visitors compared with weekday visitors, intervention parks had a greater proportion of weekend (*p* = 0.001) and morning period visitors (*p* < 0.001). The proportion of park visitors who were sedentary (62%) and engaged in vigorous physical activity (18%) was slightly higher in the intervention parks (*p* = 0.001) (Table 3).

In both the intervention and comparison parks men were more likely to engage in PA compared with women, however more so in the intervention parks (74% vs. 25%). While children were significantly more active than teens (*p* = 0.001) and adults (*p* = 0.02) in the comparison parks, there were no differences in PA between the age groups in the intervention parks. Park visitors from the comparison parks were significantly more active during the week compared to the weekend (*p* = 0.007), while no differences were observed in the intervention parks. Intervention park visitors were however more likely to be engaged in PA during early mornings, whereas the comparison parks showed no differences in PA and period of the day. Overall, park visitors in the comparison parks were 48% more likely to be engaged in PA, after controlling for significant age, gender, period of the day and period of the week (all significant variables in the model) (*p* < 0.001) (Table 4).

#### Subgroups of Intervention and Comparison Parks

When disaggregated, no demographic differences were observed between the upgraded district park (Nantes Park) and non-upgraded district park (Westridge Gardens). While the upgraded park was more likely to attract morning visitors (*p* < 0.001), the non-upgraded park was more likely to attract weekend visitors (*p* < 0.001) (Table 5). Upgraded district park visitors were 44% less likely to be physically active (engaging in any PA) after adjusting for age, gender, temperature and wind (OR = 0.56, 95% CI 0.46–0.69, *p* < 0.001) (Table 5).

Between the three newly developed Smart Parks and the three control parks, Smart Parks attracted a significantly greater proportion of children under 12 years (48.8% vs. 16.8%, *p* < 0.001), visitors in the early afternoon (23% vs. 6.9%, *p* < 0.001) and weekend visitors (47.7% vs. 18.3%, *p* = 0.03). There were no gender differences. Males were more likely to be physically active than females in the Smart Parks (*p* < 0.001) compared with the non-upgraded community parks. Unlike the district parks, community Smart Park visitors were 42% more likely to engage in PA compared with the control parks after adjusting for gender, period of the day and week and wind (OR = 1.42, 95%CI 1.16–1.74, *p* = 0.001) (Table 5).

When comparing the three newly developed, smaller-sized Smart parks with the larger upgraded district park, Smart parks had a significantly greater proportion of younger visitors (<12 years) (69% vs. 43%), lower proportion of adults (11% vs. 34%) (*p* < 0.001) and male visitors (62% vs. 55%, *p* < 0.001) than the upgraded district park. Smart park visitors were 70% more likely to engage in PA, after controlling for gender, period of the day and temperature (OR = 1.70, 95%CI = 1.42–2.03, *p* < 0.001).

### 3.3. Integrated Recreational/Regional Parks (Objective 2)

A total of 2135 park visitors were counted in 22 target areas in Green Point Park, the high-income regional park, amounting to an average of 112 visitors per hour in the week and 177/h on the weekend. In Valhalla Park Family Recreation Centre, the low-income regional park, 1111 visitors were counted in 19 target areas, amounting to an average of 67 visitors per hour in the week and 82/h. on the weekend, approximately half the number of visitors in Green Point Park. Table 6 describes the associations between observed park visitor characteristics in the two parks. Women were more likely to visit the high-income park, whereas men were more likely to visit the low-income park (*p* < 0.001). While children constituted the majority of park visitors in the low-income park (61.9%), the number of children and adult park visitors observed in the high-income park were similar (45.4% and 49.5%, respectively). The low-income park had a higher proportion of teenagers compared to the high-income park (22.6% and 5.1%, respectively). People visited both parks more often on weekends than on weekdays, however more so in the high-income park (*p* < 0.001). Both parks had higher number of observed park visitors in the early afternoon, followed by the late morning period.

Table 7 provides the odds ratios for participating in any PA in the two upgraded regional parks. In the high-income park, males (*p* < 0.001) and children (*p* < 0.001) showed the greatest odds of being active, while the odds of observing PA were highest in the early mornings (*p* < 0.001) despite only 1% of visitors during this period. Private exercise classes with a trainer took place during this time. In the low-income park, there were no differences in PA according to gender, age group or period of the day, except teens who were significantly less active compared to children (*p* < 0.05). Furthermore, park visitors were significantly more active during the week, compared with the weekend (*p* = 0.002). Overall, park visitors in the high-income park were 79% less likely to be engaged in any PA compared with visitors in the low-income park. Somewhat paradoxically, the odds of PA decreased by 9% for every additional sport or recreational facility within 1 mile (1.6 km) surrounding the park (OR = 0.91, 95% CI 0.90–0.93; *p* < 0.001). In this case, the high-income park had four times more surrounding facilities than the low-income comparison park (12 vs. 3).

### 3.4. Target Area Features, Park Use and Physical Activity (Objective 3)

#### 3.4.1. Intervention and Control Parks

**Figure 2:**
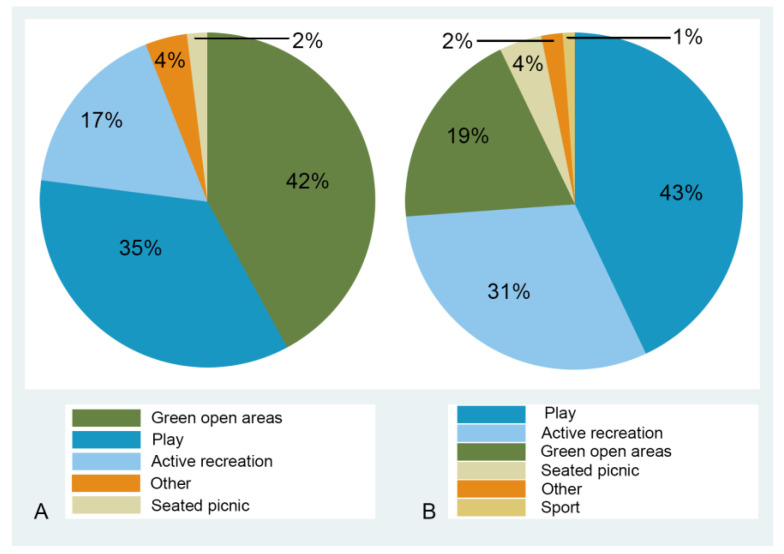
Use of park facilities in (**A**) comparison parks (*n* = 1432) and (**B**) intervention parks (*n* = 2519).

In relation to the use of and physical activity in the park target areas in the four intervention and four control parks, green open areas and play areas accounted for the most visited target areas in the comparison parks (42% and 35%, respectively) (Figure 2A). Less than 20% (*n* = 287) of park visitors were observed in active recreation areas, the majority (*n* = 207) of which were observed in the skate park in Westridge Gardens. In the intervention parks (Figure 2B), the proportion of park users utilising green open spaces was lower (19%) with a higher proportion of visitors in the play areas (43%) and active recreation and sport facilities (31%), such as the multi-purpose courts, skate park and outdoor gyms.

Like target area features in the subgroups of parks were compared. In the upgraded district park, use of and PA was significantly greater in play areas compared with the non-upgraded district park (use = 74% vs. 26%, *p* < 0.001; PA = 67% vs. 33%, *p* = 0.001, respectively), while lower in the green open spaces (use = 38% vs. 62%, *p* < 0.001; PA = 26% vs. 74%, *p* < 0.001) and skate park (use = 47% vs. 53%, *p* < 0.001; PA = 37% vs. 63%, *p* = 0.003).

In the Smart parks, play areas attracted significantly more users than the control parks (63% vs. 37%, *p* < 0.001), while there was a slightly higher proportion in the Smart parks green open spaces (55% vs. 45%). There was no difference in PA in both the play areas and green open spaces. When comparing the Smart parks playground areas to Nantes Park playground area, PA in the Smart parks was significantly higher (58% vs. 42%, *p* = 0.002).

#### 3.4.2. Integrated Recreational/Regional Parks

**Figure 3:**
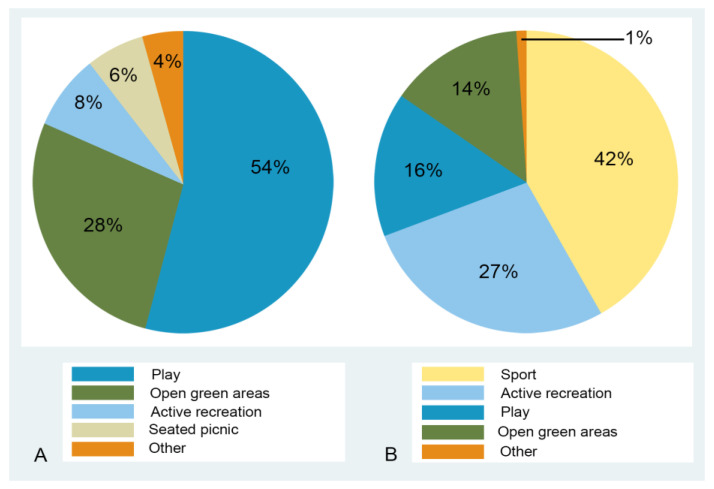
Use of park facilities in (**A**) Green Point Park (*n* = 2135) and (**B**) Valhalla Park family Recreation Centre (*n* = 1111).

In the high-income park, as play areas and green open areas formed the two main areas of the park, these target areas attracted the highest number of visitors (54% and 28%, respectively). The only active recreation area was the outdoor gym, which accounted for less than 10% of observed visitors (*n* = 171) (Figure 3A). In the low-income park, sport and active recreation areas attracted nearly 70% of visitors, with 16% (*n* = 179) observed in play areas. Only 14% were counted in green open areas (Figure 3B).

#### 3.4.3. Target Area Features, Park Users and Physical Activity

The logistic regression model (Table 8) estimated the association between target area features (independent variables) with the likelihood of subgroups of gender and age being observed in that location of the park. Features such as play areas and seated picnic areas were significantly associated with park use among females, while active recreation and sport facilities, particularly the skate park, multi-purpose courts and soccer area were significantly associated with male use. Furthermore, there was a higher odds of observing females when parks provided separate play areas for younger and older children. While children were associated with the use of play areas and netball/basketball courts, active recreation, sport and green open areas were associated with a greater odds of use among teens. Park facilities and areas related to adult use included the outdoor gym (OR = 2.19), green open areas (OR = 1.70) and seated picnic areas (OR = 3.26).

In Table 9, the logistic regression model estimated the association between target area features (independent variables) with the likelihood of subgroups of gender and age being observed being physically active in that location of the park. Among females, the netball/basketball courts were the only park facilities positively associated with physical activity. Males in turn had a greater odds of physical activity in nearly all areas of the park, particularly the soccer field (OR = 3.26), multi-purpose courts (OR = 1.95), play areas (OR = 1.63) and green open areas (OR = 1.60). Children were physically active in play areas, green open spaces (OR = 2.24), the soccer field (OR = 1.97) and skate parks (OR = 1.73). Two park facilities were significantly related to park use in teens: the multi-purpose courts (OR = 1.79) and netball/basketball courts, where they were seven times more likely to engage in PA (OR = 7.08). Unexpectedly, the only area where adults had a significant odds of engaging in PA were the netball/basketball courts (OR = 14.46).

## 4. Discussion

The provision of public parks may serve as a fundamental means of improving health outcomes and quality of life in disadvantaged communities. In South Africa, the renewal and development of social public facilities, and in particular public parks, have become a key priority of local governments, creating the need and opportunity for evaluation. In this observational study, the comparison between upgraded, newly developed and non-upgraded parks in low-middle income settings, and between two large parks in different income settings, provides insight to the use of public parks and park-based physical activity in Cape Town. Overall, our study observed a greater number of males than females in nine parks except the park situated in the high-income area. Similarly, males were more likely, in certain parks, to be more physically active. As expected in parks with playgrounds, the majority of visitors observed were children. However, it is concerning that only 13 older adults were observed. Parks were visited more frequently on weekends and in the early afternoons, and while the majority of park visitors were observed in sedentary activities, certain parks and park features were associated with more visitors engaged in physical activity. What is of special interest is how youth interact with the different park features. This discussion explores the park user characteristics among the parks and the features associated with greater use and physical activity, and aims to answer the question: If you build it, will they come?

### 4.1. Use, Physical Activity and Features in the 10 Parks

#### 4.1.1. Gender, Physical Activity and Park Features

As supported by numerous studies [44,45,46], gender differences exist in park use and physical activity. In our study, we found more males physically active in relation to females in the larger district parks and smaller Smart Parks, however these differences were not significant in the non-upgraded community parks and the lower income regional park (Valhalla Park Family Recreation Centre). Associations with the target area features revealed that more males were observed in active recreational areas, such as the skate park, multi-purpose courts and soccer field, and active in the play areas (younger boys), multi-purpose courts and soccer field. The two district parks (upgraded and non-upgraded) and Smart Parks contained these features while the lower income regional park was the only park with a full-sized Astroturf soccer field. Among females, while play areas were associated with use, the netball/basketball courts were the only park facilities positively and significantly associated with physical activity. In younger girls, Cohen et al. [46] found playgrounds and basketball courts associated with higher non-school physical activity.

Due to the burden of lifestyle-related NCDs which account for 30% of deaths in women in the Western Cape district [47], access to and opportunities for recreation to improve female’s (girls and adult women) health is pertinent. Therefore, park design and features such as the inclusion of family orientated or intergenerational play equipment, walking paths around play areas, and multi-purpose courts with netball court markings may be key to encourage park use and physical activity among females [28]. Structured active recreation programmes have also shown to encourage greater park use and activity [48]. While these features may not attract all females, park features conducive for picnicking, socialising and relaxation may encourage more female patrons [28].

#### 4.1.2. Age Group, Physical Activity and Park Features

While children are synonymous with the use of parks and playgrounds, of interest is its use by youth. In our study, nine of the ten parks service suburbs experiencing high crime and unemployment rates (www.crimestatssa.com). For youth living under these conditions, parks and recreational facilities offer an opportunity to engage in constructive activities during after-school hours and weekends. In the lower income regional park and the Smart parks where active recreation and sport facilities are provided, we observed a higher number of teenagers. In the Smart parks, we observed no significant differences in PA between children and teenagers. Knapp found that park use in teenagers were associated with the number of activity settings as well as attractiveness [49].

When exploring the park features, the skate park had the highest odds of use among teenagers, and the multi-purpose and netball/basketball courts with physical activity. We found children more likely to be physically active in the two skate parks rather than teenagers. In one of our parks, the non-upgraded district park with a long-established skate park, children used this space for biking, skating, rollerblading and scooter riding. While teens used this space for skating, they also sat on the stadium-like tiered seating overlooking the skate park. Numerous studies support increased use and physical activity by teenagers after the installation of active recreation features. In a low-income neighbourhood in Denver, Colorado, U.S., the proportion of park visits by teenagers doubled (14.4% to 28.1%) after the renovation of a recreational park that included the development of a multi-purpose playing field, ball courts and play area. The majority of teenagers is this study were attracted to ball courts where most of them engaged in PA [50]. Lindberg and Schipperijn [51] found that teenagers who use sport and active recreation features such as a skate park, soccer field, basketball court and multi-court were mostly physically active. As Cohen suggests, the nature of facilities added may influence use in this age group [52]. However, park features alone are not sufficient to attract teenagers. McCormack et al. [28] found that youth who visited parks appeared to visit a central secluded area to ‘hang-out’ and socialise when unaccompanied by adults. Furthermore, in high-school youths, a greater likelihood of park use was associated with perceptions of greater park availability, quality (*p* ≤ 0.01), and use by friends (*p* ≤ 0.001) [25]. Hence, the park’s social attractiveness may also appear to support use. To encourage park use, social interaction and physical activity in teenagers, a potential design solution is the creation of ‘activity hubs’ which place facilities for physical activity in close proximity to each other. For example, placing multi-purpose courts, netball/basketball courts and skate parks within close proximity, and provide age-appropriate programming.

Finally, we further showed that youth were significantly less active than children in the high-income regional park and the control parks. It is important to note that visitation by teenagers to the high-income regional park, with close proximity to the sea, were more likely to be linked to “special occasions” or family outings such as family picnics or birthday celebrations, rather than fitness or physical activity opportunities. This may explain, at least in part, the lower levels of physical activity seen in high-income regional park users in comparison to the low-income park users.

It is concerning that fewer older adults were observed in our study (*n* = 13) in relation to the younger age groups. This is lower than that observed in a national study in the US where 4% of park visitors were older adults [53]. In our study, 10 of the 13 senior adults were observed in the larger intervention parks. In these parks, six seniors were observed in playground areas, highlighting their role in child supervision. Only three were observed in active recreation areas (outdoor gym and soccer field) while the remainder in passive recreation areas such as picnic areas. Due to generally lower levels of physical activity in this age-group [54] efforts to promote physical activity and provide conducive environments for it are key. Improving accessibility, providing structured activities and improving opportunities for relaxation and socialising has shown to encourage the use of parks and green spaces by seniors [55,56,57]. Creating a safe environment for older adults is also a key factor in outdoor recreation [52], particularly in lower income settings experiencing a high degree of crime [15].

In our study, despite 74% of park visitors observed in play and active recreation areas, a noticeable observation was the proportion of sedentary park visitors (62%). Similarly, 63% of park visitors were observed sedentary in a large metropolitan park in Australia [33]. These findings suggest that while the traditional function of parks serve as play areas for children, parks also serve as places for relaxation and social interaction among its visitors [58].

### 4.2. Intervention and Comparison Parks

In our intervention parks (upgraded District Park and Smart parks), a greater number of visitors were observed compared with the non-upgraded parks. Numerous empirical studies evaluating the impact of park renovations have found significant increases in park use, park-based physical activity [59] as well as sedentary behaviour in comparison with control parks [29,60]. This impact has been shown to persist over time [29].

#### 4.2.1. Upgraded and Non-Upgraded District Parks

When comparing park use, physical activity and target areas features, the upgrade of the district park play area with multiple play zones was successful to attract a greater number of visitors and those engaged in PA in relation to the control park play area. Conversely, despite the development of a new skate park, the long-established skate park in the non-upgraded park attracted not only a higher number of visitors, but also more engaged in PA. Overall, the upgraded park, although receiving upgrades to enhance physical activity and play, found park visitors significantly less likely to be physically active than the non-upgraded control park. The findings show that the intervention park attracted more visitors overall, but only active behaviour in the upgraded play areas. Overall, a higher proportion of visitors were sedentary compared with the non-upgraded park (71% vs. 55%). This suggests that upgraded park visitors enjoyed socialising and relaxing in the park. The non-upgraded park however attracted a higher proportion of users and more physical activity behaviour in the green open spaces (where ball games are allowed) and skate park, accounting for the overall higher proportion of active visitors.

#### 4.2.2. Community Smart Parks

In the case of the three newly developed community Smart parks, more than double the number of visitors were observed compared with the non-upgraded community parks (1499 vs. 618 visitors), as well a greater likelihood of physical activity (48.8% vs. 16.8%). When comparing the two intervention types: smaller newly developed community Smart parks with the larger and older upgraded district park, we reported a 70% greater odds of physical activity in the Smart parks. The Smart parks in turn, attracted a significantly higher proportion of children and males, as well as a higher proportion of users and PA observed in the newly established playground areas and multi-purpose recreational facilities compared with the existing play areas of the control parks and upgraded district park.

In low-income neighbourhoods in Los Angeles, California, three newly developed and smaller community ‘pocket parks’ were compared with existing larger neighbourhood parks. While the authors reported significantly more users on average in the pocket parks, PA was found to be lower (24% vs. 40%, *p* < 0.001). Contributing factors to the lower PA include a lower proportion of children and teens (64% vs. 79%, *p* < 0.001), the observation of more females (63% vs. 56%, *p* = 0.0068), who in turn tended to be less active, the use of the picnic areas (accounting for 23% of users), as well as the limited facilities that enabled vigorous activity, such as soccer and basketball courts, when compared with the larger neighbourhood parks [61]. Conversely, our study in the smaller community parks found more males, children and users who were attracted to the active recreation areas.

The novelty of the new Smart Parks may influence the number of visitors and levels of physical activity when compared with the upgraded district park, in which renovations had occurred two years prior to our evaluation. Cohen et al. [52], after evaluating the short-term (immediately after renovation) and long term effects (after 2–3 years after renovation) in five renovated parks in San Francisco reported that increases in park use and PA were attributed mostly to the short term effects (580% increase in park use and 800% increase in MET-hours, *p* < 0.001), with slight decreases in use (−53%) and PA (−60% in MET-hours) at the second wave of evaluation (*p* < 0.05). Similarly, Veitch et al. [33] evaluating an upgraded park in a low socio-economic area in 2013, showed an increase in park use by 33% after one year but then remained stable to year two (2015). Both studies have highlighted the immediate spike in park use after the intervention then the stabilisation effect of time at year two to three. In the case of our Smart parks, the greater attendance by children and youth and the availability of opportunities for PA, such as multi-purpose courts, appears to have impacted on overall PA when compared with the older and larger intervention park. However, it is pertinent that longitudinal evaluations unpack the dynamic of use and PA over time.

### 4.3. Integrated Recreational/Regional Parks

Few studies have shown differences in park use and park-based physical activity between parks in high- and low-income settings. In our study, the high-income regional park attracted nearly twice the number of visitors than the low-income regional park. This is supported by other studies [62,63]. In Cohen et al. [63], the difference was attributed high-income parks that were nearly twice as large, offered more programmes, had more staffing and were more frequently located than low-income parks. In another low-high income park study in other U.S. cities, organised and supervised activities associated strongest with physical activity in the park [37]. Conversely, residents from higher income areas tended to make less use of their public parks than those from lower-income areas in Bloemfontein City, South Africa [64].

In our parks, a difference in visitor “reach” was observed. In the high-income park, a non-residential park, 78% of visitors resided more than 1.6 km (1 mile) from the park, whereas 82.2% of park visitors from the low-income park resided within 800 m of the park. This suggests that the high-income park attracted a variety of visitors from across the city, whereas low-income park visitors resided in the community of the park (SUN study unpublished data). The greater number of visitors and reach may be attributed to the aesthetic nature of the park, the park’s public transport network, the number of parking bays, the positioning of the park within a larger recreation and sport precinct, and close proximity to the coastline with additional playground and green spaces, thereby attracting family groups travelling by motorised transport. Furthermore, the high-income park suburb provides a safer environment than the low-income park. To emphasise this, reported crime statistics in the high-income park police precinct report 738 vs. 8397 crimes reported in the low-income park police precinct in 2016.

Despite more park visitors at the high-income park, the low-income park attracted a higher proportion of children, teens and males, and overall, visitors had a significantly higher odds of engaging in physical activity. This was associated with active recreation and sport facilities, which attracted the majority of visitors in the low-income park (70%). In this park, there was no gender difference in PA. In low income parks in New Orleans, U.S., females were twice as likely to be active in attractive parks [49]. In the high-income park, the higher number of surrounding sport and recreation facilities in turn was associated with a lower odds of physical activity. Similarly, Kaczynski found greater land use diversity within a park’s buffer associated with a lower likelihood of park-based physical activity [65]. In neighbourhoods where competing interests for land space may prevent the development of multiple sport and recreation facilities, the inclusion of these sport and recreation features in the park design can provide neighbourhood residents with a variety of opportunities for active and passive recreation, particularly in lower-income areas where these opportunities are scarce.

### 4.4. Strengths

This is the first study in Africa to evaluate a government intervention on the development and upgrade of parks, specifically related to park use and park-based physical activity between parks of different income settings and between parks with different life-cycle stages (newly upgraded or developed and established parks). This study evaluated a natural intervention with matched control comparison parks conducting direct observation using a validated tool. Furthermore, the results of this study may serve to inform policy and planning practice and therefore have a translational impact as the government continues to develop parks and recreational facilities across the city, country and continent.

The study conducted direct observation using a validated tool to determine park use between a natural intervention (either newly developed or upgraded) and a matched control.

### 4.5. Limitations

Ethical approval for this study was granted at the start of the winter season, hence data collection was conducted between the winter and spring seasons. These results may therefore not reflect the number of park visitors and activities that would be observed in the spring and summer seasons. There are several limitations of the SOPARC tool. SOPARC does not provide the duration or frequency of physical activity as it is a snapshot of park use, hence the contribution of park-based physical activity to total physical activity cannot be deduced [40,43]. The tool does not provide an indication of an individual’s level of physical activity throughout their visit to the park; hence, a sufficient number of observations must be made at regular intervals [44]. Although training is a critical component, a degree of human error is possible during observations [8]. Related to the sample, as the larger district parks are designed to attract visitors from a greater catchment, the economic status of park visitors is unknown. As there are only two Regional (integrated recreational) parks in the city, limiting comparisons across more high-income/low-income pairs, the results must be interpreted with caution.

### 4.6. Future Direction

As the City of Cape Town continues to invest in park developments and upgrades as part of the Parks Development Policy, further investigation using a quasi-experimental design is recommended for future studies. For our study, this was not possible as there were no upcoming scheduled park upgrades at the time the study was conducted. Furthermore, conducting a longitudinal study in different seasons may detect a difference in use amongst the different parks, including trends in use, as the weather condition may be an influencing factor in this type of observational research. A longitudinal design will also detect social issues related to park vandalism and safety, as this is a common problem in parks in lower-income suburbs (personal communication, City of Cape Town). Similar to Zhang et al. [66], cross-cultural and cross-geographical studies are also proposed, particularly with other African cities, as this can allow for comparative analysis between activity types and areas, user profiles, differences in park-based physical activity, as well as the social and environmental correlates of park use. Lastly, it is proposed that future studies in African settings conduct a conditional audit of individual park features and amenities as well as intercept interviews across a variety of parks in different socio-economic settings to understand the relationship between neighbourhood deprivation, park use and physical activity.

## 5. Conclusions

A scarcity of evidence exists from low-middle-income countries on the role of public green space and parks on physical activity behaviour. This is the first study in Africa to describe park use and park-based physical activity and associate it with recreational features within the park. In our study, we conclude that improving the existing parks and establishing new ones indeed attract a greater number of visitors when compared with its controls. In line with existing evidence, intervention parks did not always result in more physical activity however. While park upgrades and developments improve access to public spaces for recreation, our study supports existing evidence [67] illustrating greater use of parks when a variety of recreational features exist, such as play, active recreation and sport facilities. Furthermore, it is imperative to design features and programmes that specifically target females as well as teenagers to enhance opportunities for physical activity and increase their time spent in park-based active recreation. In lower income communities with fewer recreational opportunities, the investment into public park facilities which incorporate a variety of opportunities for physical activity, relaxation and socialising may contribute towards improving levels of physical activity and physical and mental health outcomes. To promote and increase physical activity behaviour, it is our recommendation to the City government to invest in parks that provide a variety of active recreational opportunities, particularly as the smaller Smart parks were the likeliest to promote physical activity in lower income communities.

## Figures and Tables

**Table 1:** City of Cape Town Park categories.

Objective	Park Name	Park Category	Description
1	**Nantes Park** (intervention)	District Park	Large-scale landscaped multi-functional parks serving several surrounding communities or suburbs, approximately 50,000 people.
	**Westridge Gardens** (control)		These parks have at least one unique attraction and amenities, and are greater than 5 hectares in size. *
1	**NY144 Park**; **Manyanani Park**; **Longmead Park** (controls)	Community Park	Multi-functional public landscaped park spaces with informal and formal recreational facilities and have a minimum size of 1 hectare. They serve a population size of approximately 20,000 people from the surrounding local communities or suburbs. *
	**NY 110 Smart Park**; **Mandela Smart Park**; **Symphony Way (Blikkiesdorp) Smart Park** (interventions)	Community Smart Park (category of Community Park)	Smart Parks are community parks that aim to provide the most under-served communities with high quality, sustainable park facilities which are safe and easily accessible. Details of the Smart Parks Programme can be perused elsewhere [32]
2	**Green Point Urban Park** (intervention-high-income area)	Regional Park	Large-scale multi-functional parks, meeting the wide-ranging needs of the district or region. Preserves unique and often extensive landscapes. Serves several surrounding communities or suburbs, approximately 60,000 people.
	**Valhalla Park Family Recreation Centre** (intervention-lower-income area)		Cluster of informal and formal recreational and related facilities that may be integrated with other public facilities such as a community hall, library or health clinic.

* City of Cape Town, City Parks Classification Report, personal communication, Recreation and Parks Department, Planning and Development, 2017.

**Table 2:** Park features in each park.

INTERVENTION PARKS	CONTROL PARKS
Nantes Park	Westridge Gardens
**Pre-existing features** Play area Established trees for shade Walking paths (gravel) Extensive open green spaces Fenced **New features with upgrade** Multiple and larger play areas (rubberised surfaces) with walking paths Shaded and seated picnic area Outdoor gym Amphitheatre Skate and BMX park Newly established walking paths Upgrade of existing paths Upgrade of fencing Off-street parking area for cars and drop off zone for buses Paved entrance area, access controlled Public ablutions Historical centre	Play area (on gravel) with traditional play equipment Walking paths Skate park Established trees for shade Extensive open green spaces Adjacent to sports precinct (soccer, swimming, tennis) with different entrances Rose garden Ablutions Fenced
**NY 110 Smart Park**	**NY144 Park**
Play areas (rubberised surfaces) Walking paths Open green space 3 Multi-purpose Astroturf courts 1 Mini Astroturf Outdoor gym Seated picnic areas Fenced	Traditional play equipment Walking paths (on desire lines for thoroughfare) Open green space Not fenced
**Mandela Smart Park**	**Manyanani Park**
Play area for different age groups (rubberised surfaces) Walking paths Open green space 3 Multi-purpose courts 1 Mini Astroturf Outdoor gym Seated areas Fenced Parking area Community food garden	Asphalt basketball court Traditional play equipment Open green space Not fenced
**Symphony Way (Blikkiesdorp) Smart Park**	**Longmead Park**
Play area (rubberised surface) Seating 1 Multi-purpose court	Traditional play equipment Open green space One walking path (as a thoroughfare)

**Table 3:** Associations in observed park and user characteristics between intervention and comparison parks.

	Intervention (*n* = 2519)	Comparison (*n* = 1432)	X^2^ (do)	Cramér’s V	*p*
Sex, *n* (%)					
Female	1024 (40.7)	590 (41.2)	0.11 (1)	0.005	0.735
Male	1495 (59.3)	842 (58.8)			
Age group, *n* (%)					
Child	1474 (58.5)	694 (48.5)	50.79 (2)	0.113	<0.001
Teen	519 (20.6)	303 (21.2)			
Adult	526 (20.9)	435 (30.4)			
Females, *n* (5%)					
Child	608 (59.4)	290 (49.1)	18.55 (2)	0.107	<0.001
Teen	183 (17.9)	114 (19.3)			
Adult	233 (22.7)	186 (31.5)			
Males, *n* (%)					
Child	866 (57.9)	404 (48.0)	32.91 (2)	0.119	<0.001
Teen	336 (22.5)	189 (22.4)			
Adult	293 (19.6)	249 (29.6)			
Day of week, *n* (%)					
Weekday	897 (35.6)	435 (30.4)	11.18 (1)	0.053	0.001
Weekend	1622 (64.4)	997 (69.6)			
Period of day, *n* (%)					
Morning	157 (6.2)	59 (4.1)	75.92 (3)	0.139	<0.001
Late morning	582 (23.1)	189 (13.2)			
Early afternoon	794 (31.5)	480 (33.5)			
Late afternoon	986 (39.2)	704 (49.2)			
Activity level, *n* (%)					
Sedentary	1563 (62.1)	850 (59.4)	14.43 (2)	0.06	0.001
Walking	507 (20.1)	360 (25.1)			
Vigorous	449 (17.8)	222 (15.5)			

**Table 4:** Odds ratios for any PA associations between observed park visitation characteristics and PA between district and community intervention and comparison parks.

	Intervention		Comparison	
	OR for PA (95% CI)	*p*	OR for PA (95% CI)	*p*
Sex				
Female (ref)	1.00		1.00	
Male	1.74 (1.47, 2.06)	<0.001	1.25 (1.01, 1.55)	0.04
Age group				
Child (ref)	1.00		1.00	
Teen	0.93 (0.75, 1.14)	0.465	0.62 (0.47, 0.83)	0.001
Adult	0.91 (0.74, 1.11)	0.351	0.74 (0.58, 0.94)	0.016
Females				
Child (ref)	1.00		1.00	
Teen	0.70 (0.49, 1.02)	0.061	0.76 (0.49, 1.18)	0.220
Adult	0.73 (0.52, 1.01)	0.060	0.72 (0.50, 1.04)	0.077
Males				
Child (ref)	1.00		1.00	
Teen	1.03 (0.81, 1.32)	0.799	0.65 (0.47, 0.91)	0.012
Adult	1.14 (0.88, 1.47)	0.326	0.97 (0.72, 1.31)	0.839
Day of the week				
Weekend day (ref)	1.00		1.00	
Week day	1.16 (0.98, 1.37)	0.078	1.37 (1.09, 1.72)	0.007
Period of the day				
Morning (ref)	1.00		1.00	
Late-morning ^1^	0.53 (0.37, 0.78)	0.001	0.82 (0.45, 1.52)	0.535
Afternoon ^2^	0.59 (0.42, 0.84)	0.003	0.97 (0.55, 1.71)	0.926
Late-afternoon ^2^	0.47 (0.33, 0.65)	<0.001	0.72 (0.42, 1.25)	0.243
Park by intervention				
Intervention (ref)	1.00		-	
Comparison	1.48 (1.30, 1.69) ^3^	<0.001	-	

^1^ Adjusted for average minimum temperature (9 °C); ^2^ Adjusted for average maximum temperature (22 °C). ^3^ Adjusted for gender, period of the day and week.

**Table 5:** Odds ratios for associations between observed park visitation characteristics and PA between intervention and comparison parks.

	Upgraded District Park		Non-Upgraded District Park		Smart Parks		Non-Upgraded Community Parks	
	OR for PA (95% CI)	*p*	OR for PA (95% CI)	*p*	OR for PA (95% CI)	*p*	OR for PA (95% CI)	*p*
Sex:								
Female (ref)	1.00		1.00		1.00		1.00	
Male	1.39 (1.06, 1.83)	0.019	1.63 (1.23, 2.16)	0.001	1.90 (1.53, 2.35)	<0.001	0.92 (0.66, 1.29)	0.645
Age group:								
Child (ref)	1.00		1.00		1.00		1.00	
Teen	0.98 (0.62, 1.40)	0.937	2.08 (1.42, 3.03)	<0.001	0.98 (0.75, 1.28)	0.895	1.12 (0.68, 1.87)	0.646
Adult	0.89 (0.65, 1.21)	0.445	1.91 (1.39, 2.61)	<0.001	0.82 (0.87, 1.67)	0.294	0.99 (0.65, 1.50)	0.968
Females:								
Child (ref)	1.00		1.00		1.00		1.00	
Teen	1.60 (0.86, 2.93)	0.131	2.44 (1.30, 4.58)	0.006	1.57 (0.97, 2.53)	0.065	0.75 (0.34, 1.63)	0.476
Adult	2.01 (1.24, 3.27)	0.005	2.26 (1.41, 3.62)	0.001	0.87 (0.52,1.47)	0.611	0.81 (0.39, 1.67)	0.570
Males:								
Child (ref)	1.00		1.00		1.00		1.00	
Teen	0.72 (0.45, 1.14)	0.016	2.03 (1.25, 3.30)	0.004	0.78 (0.56, 1.08)	0.134	1.64 (0.80, 3.30)	0.172
Adult	0.47 (0.31, 0.73)	0.001	1.65 (1.08, 2.54)	0.021	0.76 (0.51, 1.17)	0.219	1.09 (0.65, 1.82)	0.726
Day of the week:								
Weekend day (ref)	1.00		1.00		1.00		1.00	
Week day	1.11 (0.84, 1.47)	0.439	1.46 (1.06, 2.00)	0.021	1.28 (1.03, 1.59)	0.026	1.48 (1.05, 2.08)	0.022
Period of the day:								
Morning (ref)	1.00		1.00		1.00		1.00	
Late-morning ^1^	2.42 (1.19, 4.92)	0.015	0.74 (0.11, 5.02)	0.760	1.93 (1.20, 3.12)	0.006	1.45 (0.73, 2.91)	0.219
Afternoon ^2^	2.18 (1.19, 3.98)	0.011	3.33 (0.63, 17.58)	0.157	1.42 (0.90, 2.24)	0.127	0.84 (0.41, 1.73)	0.651
Late-afternoon ^2^	2.26 (1.26, 4.05)	0.006	3.27 (0.62, 17.18)	0.162	1.95 (1.25, 3.04)	0.003	1.51 (0.79, 2.91)	0.209
Park by intervention:								
Comparison (ref)	1.00		-		1.00		-	
Intervention	0.56 (0.46, 0.69) ^3^	<0.001	-		1.42 (1.16–1.74) ^4^	<0.001	-	

^1^ Adjusted for average minimum temperature (9 °C); ^2^ Adjusted for average maximum temperature (22 °C). ^3^ Adjusted for age, gender (male), temperature and wind; ^4^ Adjusted for gender (male), period of day and week, and wind.

**Table 6:** Associations in observed park and user characteristics between Green Point Park and Valhalla Park Family Recreation Centre.

	Green Point Park (*n* = 2135)	VPFRC (*n* = 1111)	X^2^ (df)	Cramér’s V	*p*
Sex, *n* (%)					
Female	1205 (56.4)	320 (28.8)	224.1 (1)	−0.26	<0.001
Male	930 (43.6)	791 (71.2)			
Age group, *n* (%)					
Child	969 (45.4)	688 (61.9)	464.1 (2)	0.38	<0.001
Teen	109 (5.1)	251 (22.6)			
Adult	1057 (49.5)	172 (15.5)			
Females, *n* (%)					
Child	461 (38.3)	232 (72.5)	229.7 (2)	0.39	<0.001
Teen	73 (6.0)	59 (18.4)			
Adult	671 (55.7)	29 (9.1)			
Males, *n* (%)					
Child	508 (54.6)	456 (57.6)	211.3 (2)	0.35	<0.001
Teen	36 (3.9)	192 (24.3)			
Adult	386 (41.5)	143 (18.1)			
Day of week, *n* (%)					
Weekday	897 (42.0)	534 (48.1)	10.8 (1)	0.06	0.001
Weekend	1238 (58.0)	577 (51.9)			
Period of day, *n* (%)					
Morning	18 (0.9)	39 (3.5)	46.9 (3)	0.12	<0.001
Late morning	589 (27.6)	231 (20.8)			
Early afternoon	1079 (50.5)	618 (55.6)			
Late afternoon	449 (21.0)	223 (20.1)			
Activity level, *n* (%)					
Sedentary	1522 (71.3)	577 (51.9)	143.8 (2)	0.21	<0.001
Walking	399 (18.7)	275 (24.8)			
Vigorous	214 (10.0)	259 (23.3)			

**Table 7:** Odds ratios for associations between observed park visitation characteristics and PA between Green Point Park and Valhalla Park Family Recreation Centre.

	Green Point Park		VPFRC	
	OR (95% CI)	*p*	OR (95% CI)	*p*
Sex:				
Female (ref)	1.00		1.00	
Male	1.42 (1.18, 1.72)	<0.001	0.97 (0.76, 1.27)	0.874
Age group:				
Child (ref)	1.00		1.00	
Teen	0.40 (0.25, 0.65)	<0.001	0.74 (0.55, 0.99)	0.05
Adult	0.36 (0.29, 0.44)	<0.001	0.83 (0.60, 1.17)	0.291
Day of the week:				
Weekend day (ref)	1.00		1.00	
Week day	0.99 (0.82, 1.20)	0.958	1.46 (1.16, 1.86)	0.002
Period of the day:				
Morning (ref)	1.00		1.00	
Late-morning ^1^	0.03 (0.004, 0.22)	0.001	0.76 (0.37, 1.55)	0.453
Afternoon ^2^	0.02 (0.003, 0.15)	<0.001	0.54 (0.28, 1 05)	0.071
Late-afternoon ^2^	0.03 (0.003, 0.20)	<0.001	0.59 (0.28, 1.22)	0.155
Park by SES:				
Low VPFRC	1.00		-	
High GPP	0.21 (0.14, 0.32) ^3^	<0.001	-	

^1^ Adjusted for average minimum temperature (9 °C); ^2^ Adjusted for average maximum temperature (22 °C). ^3^ Adjusted for age, gender, period of the week, temperature.

**Table 8:** Association between target area features and park use by demographic characteristics.

Independent Variables	Gender				Age Group					
(Referent)	Female		Male		Child (≤12 Years)		Teen (13–20 Years)		Adult (≥21 Years)	
	OR	95% CI	OR	95% CI	OR	95% CI	OR	95% CI	OR	95% CI
Play areas:										
Play areas for younger	**2.62**	**2.20–3.12**	**0.38**	**0.32–0.46**	**0.79**	**0.67–0.94**	0.79	0.62–1.01	1.10	0.91–1.32
Play areas for older	**2.08**	**1.82–2.38**	**0.48**	**0.42–0.55**	1.08	0.95–1.24	**0.19**	**0.14–0.26**	0.96	0.82–1.11
Play areas for all ages	**1.25**	**1.11–1.41**	**0.80**	**0.71–0.90**	**3.17**	**2.78–3.62**	**0.41**	**0.33–0.51**	**0.54**	**0.47–0.63**
Play areas combined	**2.25**	**2.05–2.48**	**0.44**	**0.40–0.49**	**1.90**	**1.72–2.09**	**0.31**	**0.26–0.36**	**0.70**	**0.63–0.78**
Active recreation:										
Skate park	**0.26**	**0.20–0.32**	**3.89**	**3.09–4.88**	**0.65**	**0.54–0.79**	**3.39**	**2.76–4.17**	**0.61**	**0.49–0.76**
Multi-purpose courts	**0.16**	**0.13–0.22**	**5.91**	**4.53–7.72**	1.00	0.83–1.22	**1.71**	**1.37–2.15**	**0.65**	**0.49–0.84**
Outdoor gym	1.08	0.87–1.34	0.93	0.75–1.15	**0.49**	**0.39–0.62**	0.78	0.57–1.01	**2.19**	**1.74–2.74**
Sport:										
Soccer	**0.12**	**0.08–1.17**	**8.50**	**5.89–12.26**	1.23	0.99–1.52	**1.50**	**1.16–1.94**	**0.71**	**0.55–0.92**
Netball/basketball courts	0.84	0.54–1.32	1.18	0.76–1.85	**3.09**	**1.84–5.20**	0.74	0.38–1.43	**0.33**	**0.16–0.66**
Green open areas	1.03	0.93–1.15	0.97	0.87–1.08	**0.48**	**0.43–0.54**	**1.63**	**1.42–1.87**	**1.70**	**1.52–1.91**
Seated picnic areas	**2.04**	**1.57–2.64**	**0.49**	**0.38–0.64**	**0.46**	**0.35–0.60**	0.72	0.49–1.06	**3.26**	**2.48–4.28**

All odds ratios have been adjusted for park size; bold odds ratios and confidence intervals indicate significant associations (*p* < 0.05).

**Table 9:** Association between target area features and park-based physical activity (PA) by demographic characteristics.

Independent Variable	Gender				Age Group					
(Referent)	Female		Male		Child (≤12 Years)		Teen (13–20 Years)		Adult (≥21 Years)	
	OR	95% CI	OR	95% CI	OR	95% CI	OR	95% CI	OR	95% CI
Play areas:										
Play areas for younger children	0.69	0.48–01.00	1.44	1.00–2.10	**1.84**	**1.28–2.65**	0.7	0.40–1.24	**0.52**	**0.34–0.81**
Play areas for older children	**0.61**	**0.47–0.79**	**1.63**	**1.26–2.12**	**2.34**	**1.79–3.07**	1.39	0.74–2.61	**0.33**	**0.24–0.47**
Play areas for all ages	**0.70**	**0.56–0.89**	**1.42**	**1.13–1.79**	**1.34**	**1.03–1.73**	1.00	0.66–1.51	**0.69**	**0.51–0.93**
Play areas combined	**0.65**	**0.56–0.76**	**1.50**	**1.31–1.79**	**1.81**	**1.54–2.13**	0.93	0.70–1.25	**0.49**	**0.40–0.60**
Active recreation:										
Skate park	0.95	0.62–1.46	1.05	0.68–1.61	**1.73**	**1.21–2.48**	**0.43**	**0.30–0.63**	1.41	0.95–2.10
Multi-purpose courts	**0.51**	**0.31–0.87**	**1.95**	**1.16–3.28**	**0.57**	**0.40–0.81**	**1.79**	**1.20–2.68**	1.26	0.76–2.07
Outdoor gym	1.2	0.78–1.83	0.83	0.54–1.28	1.32	0.85–2.05	0.54	0.29–1.02	1.00	0.65–1.53
Sport:										
Soccer	**0.31**	**0.12–0.82**	**3.26**	**1.22–8.69**	**1.97**	**1.26–3.08**	**0.49**	**0.28–0.88**	0.74	0.43–1.26
Netball/basketball courts	**6.97**	**2.59–18.75**	**0.14**	**0.05–0.39**	**0.08**	**0.02–0.29**	**7.08**	**1.40–35.8**	**14.46**	**1.71–122.10**
Green open areas	**0.62**	**0.51–0.76**	**1.60**	**1.32–1.95**	**2.24**	**1.84–2.73**	0.82	0.65–1.04	**0.51**	**0.41–0.62**

Bold odds ratios and confidence intervals indicate significant associations (*p* < 0.05).

## Data Availability

The data presented in this study are available on request from the corresponding author. The data are not publicly available as primary data for the purposes of this study was collected.
